# Biodiversity of cultivable *Burkholderia* species in Argentinean soils under no-till agricultural practices

**DOI:** 10.1371/journal.pone.0200651

**Published:** 2018-07-12

**Authors:** Walter Omar Draghi, Jose Degrossi, Magalí Bialer, Graciela Brelles-Mariño, Patricia Abdian, Alfonso Soler-Bistué, Luis Wall, Angeles Zorreguieta

**Affiliations:** 1 Fundación Instituto Leloir, IIBBA CONICET, Buenos Aires, Argentina; 2 Instituto de Biotecnología y Biología Molecular–CCT La Plata CONICET, Universidad Nacional de La Plata, La Plata, Argentina; 3 Facultad de Farmacia y Bioquímica, Universidad de Buenos Aires, Buenos Aires, Argentina; 4 Center for Research and Development of Industrial Fermentations, (CINDEFI, CCT-LA PLATA-CONICET), Facultad de Ciencias Exactas, Universidad Nacional de La Plata, La Plata, Argentina; 5 Departamento de Ciencia y Tecnología, Universidad Nacional de Quilmes, Bernal, Argentina; Universita degli Studi di Pisa, ITALY

## Abstract

No-tillage crop production has revolutionized the agriculture worldwide. In our country more than 30 Mha are currently cultivated under no-till schemes, stressing the importance of this management system for crop production. It is widely recognized that soil microbiota is altered under different soil managements. In this regard the structure of *Burkholderia* populations is affected by soils management practices such as tillage, fertilization, or crop rotation. The stability of these structures, however, has not been evaluated under sustainable schemes where the impact of land practices could be less deleterious to physicochemical soils characteristics. In order to assess the structure of *Burkholderia* spp. populations in no-till schemes, culturable *Burkholderia* spp. strains were quantified and their biodiversity evaluated. Results showed that *Burkholderia* spp. biodiversity, but not their abundance, clearly displayed a dependence on agricultural managements. We also showed that biodiversity was mainly influenced by two soil factors: Total Organic Carbon and Total Nitrogen. Results showed that no-till schemes are not per se sufficient to maintain a richer *Burkholderia* spp. soil microbiota, and additional traits should be considered when sustainability of productive soils is a goal to fulfil productive agricultural schemes.

## Introduction

No-tillage crop production has revolutionized modern agricultural systems mostly due to the several benefits regarding soil conservation, such increasing the amount of water holding capacity, decreasing soil erosion, or increasing the amount and variety of life in soil, among others [1]. The use of this land management scheme has risen worldwide in the last years, especially since the introduction of herbicide resistant crops, which allowed a better weed control [2]. Argentina is not the exception to this phenomenon. Nowadays, more than 30 Mha are cultivated under no-tillage, stressing the importance that this practice has on the local agricultural production system (Asociación Argentina de Productores en Siembra Directa (AAPRESID); http://www.aapresid.org.ar/superficie/).

It is recognized that soil microbiota is altered under different soil managements for crop production. Previous reports showed that agricultural practices such as fertilization, crop rotation, manure amendment or pesticide application influence the biodiversity of soil prokaryotes in their habitats [3,4,5,6]. These practices could lead to a loss of soil biodiversity, which in turn impact ecosystem’s properties that could be useful for sustainable crop production, such as disease suppressiveness, resistance and resilience to abiotic stresses, and nutrient cycling [7,8]. In this sense, the use of sustainable agricultural practices which preserve biological diversity is essential to ensure long term schemes of soil use for crop production.

The genus *Burkholderia* is a group of β-proteobacteria commonly found in nature and regularly associated to higher organisms such as plants, fungi, insects, or mammalians [9]. *Burkholderia* spp. is a common inhabitant of the soils and several species confer beneficial traits to plants improving their fitness against biotic or abiotic stressful conditions, through nitrogen fixation in legume nodules, endophytic lifestyles in roots plants, the synthesis of auxins, the modulation of ethylene levels, the biocontrol of soil-borne diseases, the induction of plant defense response, or the synthesis of siderophores [10,11,12,13]. Although several *Burkholderia* exhibit potential biological applications, some relevant species belong to the *Burkholderia cepacia* complex (BCC), a set of 20 genetically closed related species usually found in cystic fibrosis and other immunocompromised patients [14]. Interestingly, several isolates belonging to the BCC also depict plant growth promoting mechanisms, such as the nitrogen-fixing *Burkholderia vietnamiensis*, or *Burkholderia ambifaria* and *Burkholderia cepacia*, which promote plant growth through auxin synthesis or siderophore production respectively, while *Burkholderia pyrrocinia* exert biocontrol activity through the synthesis of the antifungal compound pyrrolnitrin [13,15,16,17,18,19]. Thus, the abundance and diversity of *Burkholderia* spp. species could strongly influence the soil productivity through the several biological functions that positively act on crop development.

The taxonomy of the *Burkholderia* genus is constantly revisited, and the genus could be categorized in three main clades [20,21]. Group I (BCC) is set up by species belonging to the *Burkholderia cepacia Complex*, as well as phylogenetically related species, showing pathogenic characteristics in their interaction with higher organisms. Group 2 is integrated by *Burkholderia* species commonly recognized as Plant Beneficial Environment strains (PBE), which exert plant-growth promotion or environmental beneficial effects through biological nitrogen fixation, biocontrol activity, phytoremediation, and other positive traits. The third group is composed by a group of recently described species, related to *B*. *glathei* (*Burkholderia glathei* group, BGG). Although each group was recently classified into new prokaryotes genera (PBE species belong to the *Paraburkholderia* genus [22], BGG species belong to the *Caballeronia* genus [23], while BCC species remain in the *Burkholderia* genus), some authors still disagree with that classification [21,24]. In fact, the International Committee on Systematics of Prokaryotes, Subcommittee for the Taxonomy of *Rhizobium* and *Agrobacterium* have recently stated the needed for a more conclusive approach to unravel the taxonomy of the *Burkholderia* genus [25]. Clearly, a more robust analysis is needed to define a robust taxonomy into this bacterial genus.

It is widely recognized that the land use affects the community structure of bacterial populations in agricultural soils. In particular, the presence and diversity of *Burkholderia* species were affected by agricultural practices, such as crop rotation, tillage, and plant species [26,27,28]. Furthermore, it has been shown that the diversity and richness of soil bacterial communities differed by ecosystem type, showing distinctive biogeographic patterns across different sites modulated mainly by edaphic factors [29,30]. In addition, contrasting agricultural practices (such as ploughing and no-till) exert severe effects on soil physical and chemical properties, e.g. bulk density, organic matter, or microbial biomass. However, the magnitude of the changes produced by conventional tillage cannot be easily extrapolated when analyzing no-till land uses (as in our case), mainly due to the magnitude of environmental changes imposed by intensive tillage systems that severely change soil properties and, concomitantly, the structure of bacterial population, as previously showed for the *Pseudomonas* genus [31]. In order to unraveling the influence of non-till soil management schedules on the structure of actively growing *Burkholderia* species, we performed an extensive analysis of the cultivable population of *Burkholderia* spp. on soils belonging to the main productive area of Argentina.

## Methods

### Geographical sites and soil sampling

Soil samples were collected in two consecutive years (2010 and 2011), both in mid-summer (February) and late winter (September) across a West-East transect in four different geographical sites of the central agricultural productive area of Argentina (S1 Fig): Bengolea and Monte Buey (Córdoba Province), Pergamino (Buenos Aires Province) and Viale (Entre Rios Province). The owners of the land gave permission to conduct the study on these sites.

The field study did not involve endangered or protected species. The information about soil physicochemical properties and the record of agricultural practices of the three sites in the previous five years (2004–2009) before the beginning of sampling is shown in Table 1. According to these records, three soil managements were defined: Sustainable (Good) Agricultural Practices (GAP) and Non Sustainable (Bad) Agricultural Practices (BAP). GAP schemes are represented by crop management systems under sustainability principles: no-till seeding soils subjected to intensive crop rotation, balanced nutrient replacement, minimized agrochemical use (herbicides, insecticides and fungicides) and presence of winter cover crops in the rotation schedules. BAP schemes correspond to agricultural management systems, also under no-till methods, but showing higher crop monoculture (represented mainly by soybean crop), low nutrient replacement and higher doses of agrochemical applied on crops (herbicides, insecticides and fungicides). Finally, Natural Environments (NE) used as references, are represented by grassland landscapes that were not cultivated at least in the last 30 years. NE sites were selected in an area of approximately 1 hectare close to the cultivated plots (less than 5 km apart).

**Table 1:** Soil and environmental characteristics at each sampling location.

Site	Bengolea		Monte Buey			Pergamino		Viale	
Longitude	63 37´53´´W		60 27´06´´W			60 33´57´´W		59 40´07´´W	
Latitude	33 01´31´´S		32 58´14´´S			33 56´36´´S		31 52´59´´S	
Soil classification	EnticHaplustoll		TypicArgiudol			TypicArgiudoll		VerticArgiudoll	
Soil Texture	Sandy loam		Silt loam			Silt loam		Silty clay loam	
Mean Annual Precipitation (mm)	884,62		929,9			1002.7		1165.8	
Mean Annual Temperature (°C)	17		17			16.7		18.3	
Altitude (m)	221 m		111 m			67 m		66 m	
Treatments	GAP	BAP	GAP	BAP		GAP	BAP	GAP	BAP
No-tillage (%)	100	80	100	100		100	100	100	100
Soybean: maize ratio (%) ^a^	1.5	4	0.67	4		1.5	5	1.5	4
Winter with wheat (%)^b^	60	40	60	20		40	0	40	20
Winter with cover crops (%) ^c^	20	0	40	0		0	0	20	0
Rotation Index (Nr. Crops/year)	1.67	1.33	1.8	1.17		1.33	1	1.5	1.17
Herbicide (L/ha) ^d^	27.7	43.8	25.2	38.9		29.3	46.5	34.5	43.1
Soybean yield (kg.ha^-1^)	3067	2775	3167	2675		2933	2825	3000	1805
Maize yield (kg.ha^-1^)	10500	2700	12550	8000		9500	–	7030	3450

Geographical, soil, and climate properties at each site are shown, as well as crop management characteristics that define treatments (GAP and BAP)

a. Number of soybean cycles to number of maize cycles over the last 5 years.

b. Percentage of winters that wheat was planted as a winter crop.

c. Percentage of winters that a cover crop (*Vicia* spp.,*Melilotus alba* or *Lolium perenne*) was planted. Cover crops were chemically burned before summer crops are planted.

d. Calculated as liters of low-toxicity herbicides plus liters of moderate-toxicity herbicides weighted by two. Toxicity was defined according to EPAToxicity Categories. Units: total liters overs 5 years.

Top soil samples (0–10 cm) were collected between sowing lines in triplicates for each treatment-site in three 5-m^2^ sampling points separated at least 50 m from each other. Each replicate sample of the top 10 cm of mineral soil was collected as a composite of 16–20 randomly selected subsamples. These subsamples were combined and homogenized in the field, transported to the laboratory and stored at 4°C until processing.

### Determination of *Burkholderia* population size in soils

One gram of moist soil was suspended in sterile saline solution (0.85% NaCl) to a final volume of 10 ml. Samples were vortexed for 1 min and shaked for 30 minutes at 280 rpm and 28°C. Then, samples were immersed for 1 min in a sonication bath (40 kHz, 160 W, Testlab TB04, Argentina) and centrifuged for 5 minutes at 500 rpm. 10-fold serial dilutions were plated on PCAT (*Pseudomonas cepacia*, azelaic acid, tryptamine) medium and incubated for 5–7 days at 28º C. PCAT medium was formulated according to Burbage and Sasser but using citrulline (200 mg L^-1^) instead of tryptamine as the main nitrogen source [32]. PCAT was supplemented with cycloheximide (200 mg L^-1^) and crystal violet (2 mg L^-1^) to inhibit the growth of eukaryotic microorganisms and Gram-positive bacteria respectively [33]. To estimate the soil dry weight, 1 ml of soil suspension in triplicate were dried at 100°C until constant weight. Dry weight soil was calculated by the gravimetric method. *Burkholderia* spp. population counting was statistically analyzed in a mixed-model ANOVA (General Lineal Mixed Model), with treatments as fixed effect and sites as random effects, using the INFOSTAT package software [34]. Methods for Total Heterotrophes quantification were previously described by Agaras et al (2014). Shortly, soil suspensions were plated in triplicate on 1/10 (tryptone-soy agar, Biokar) to count total heterotrophic mesophilic bacteria (TH). Media was supplemented with cycloheximide (100μg/ml) to inhibit growth of fungi and yeasts. Colony counts were done after 48 h of incubation at 28°C.

### Identification of *Burkholderia* species through *recA* sequencing and multilocus sequencing typing (MLST)

From each soil sample, 25 colonies from PCAT medium were transferred to Tryptone Soybean Agar medium (TSA) and incubated for 48 hs at 28°C (25 c.f.u. per soil samples (*n* = 4), 3 treatments, 4 sampling dates, *n* = 1200). A loop of bacteria from each colony was suspended in 50 µL of sterile deionized water and heated for 15 minutes at 95° C. These samples were frozen at -20°C until PCR amplification.

Genus-specific PCR of *recA* gene fragment was performed using primers Bur3 and Bur4 as previously described by Payne and col. [35]. The reaction mixture contained 1 U Taq polymerase (PBL, Productos Bio-Lógicos®, Argentina), 250 µM of each deoxynucleoside triphosphate, 1x PCR buffer, 1.5 mM MgCl_2_, 10 pmol of each appropriate oligonucleotide primer, and 2 to 4 µL of the DNA containing solution, in a 25 µl final reaction volume. Cycling was carried out in a BioRad thermal cycler (Bio-Rad Laboratories, Hemel Hempstead, UK). Approximately 2–3 µL of each PCR product was visualized by 1% agarose gel electrophoresis.

DNA fragments were purified and sequenced on both strands with the same set of primers used in the PCR amplification by the Sanger's dideoxy chain termination method (http://www.macrogen.com). Raw sequences from both strands of the PCR products were aligned, and a consensus sequence was derived using ClustalW [36]. For the MLST analysis, DNA samples from the selected strains were used. The primers and protocols used for DNA amplification of the seven genes was described by Spilker and col. [37]. DNA fragments were visualized and sequenced as previously described.

### Phylogenetic reconstruction and species/taxon assignments of isolates

For phylogenetic tree construction, novel and selected *recA* reference sequences from *Burkholderia spp*. type strains were aligned using ClustalW, and the phylogenetic tree was inferred by the Neighbor-Joining method, using Tamura 3-parameter model as implemented in the MEGA 5 package [38]. A bootstrap confidence analysis was performed with 1000 replicates. Isolates were assigned to a *Burkholderia spp*. “like” taxon when their phylogenetic position clustered in a highly supported clade (≥ 90% bootstrapped values) with the *recA* sequence from the type strains. Multilocus sequencing typing of random-selected strains was performed as previously described [39]. Concatenated sequences of selected strains and *Burkholderia spp*. type strains obtained from the public database (http://pubmlst.org/bcc/) were aligned using ClustalW, and the phylogenetic tree was inferred by the Neighbor-Joining method, using Tamura 3-parameter model as implemented in the MEGA 5 package [38]. A bootstrap confidence analysis was performed with 1000 replicates.

### Diversity index determination and statistical analysis

To determine the variation in the diversity of *Burkholderia* spp. populations across contrasting agricultural managements, α-diversity indexes (Shannon and Simpson Indexes) were determined through EstimateS software [40]. 100 randomizations were run for all tests. In order to increase the sample size and the power of statistical tests, samples were analyzed using agricultural managements as the main factor, as described by Figuerola et al [41] and Agaras et al [31]. The evolution of diversity indexes across treatments was analyzed through linear regression and adjusted by least square model, as implemented in GraphPrism software.

### Multivariate analysis of environmental variables

The relationship between soil environmental variables and the diversity of *Burkholderia* spp. community was assessed using the Canonical Correspondence Analysis (CCA) [42], as implemented in Past Software [43]. Physicochemical data comprised total organic carbon (TOC), total nitrogen (Nt), extractable phosphorus (Pe), pH, and soil humidity, as determined by Duval et al [44].

## Results

### Quantification of total *Burkholderia* population in soils

To estimate the population size of cultivable *Burkholderia spp*. in soils subjected to different agricultural managements, we essentially followed the procedure described by Pallud and col. [33], by plating the soil samples on a semi-selective medium (PCAT) followed by PCR amplification of the *recA* amplicon in *Burkholderia* colonies using primers (Bur3-Bur4), which have been shown to be specific for the detection of the entire *Burkholderia* genus [35]. Based on the presence of the specific *recA* amplicon, an average of 47.5% (*n* = 571) of the total isolates analyzed by PCR (*n* = 1200) were recognized as *Burkholderia* isolates.

**Fig 1:**
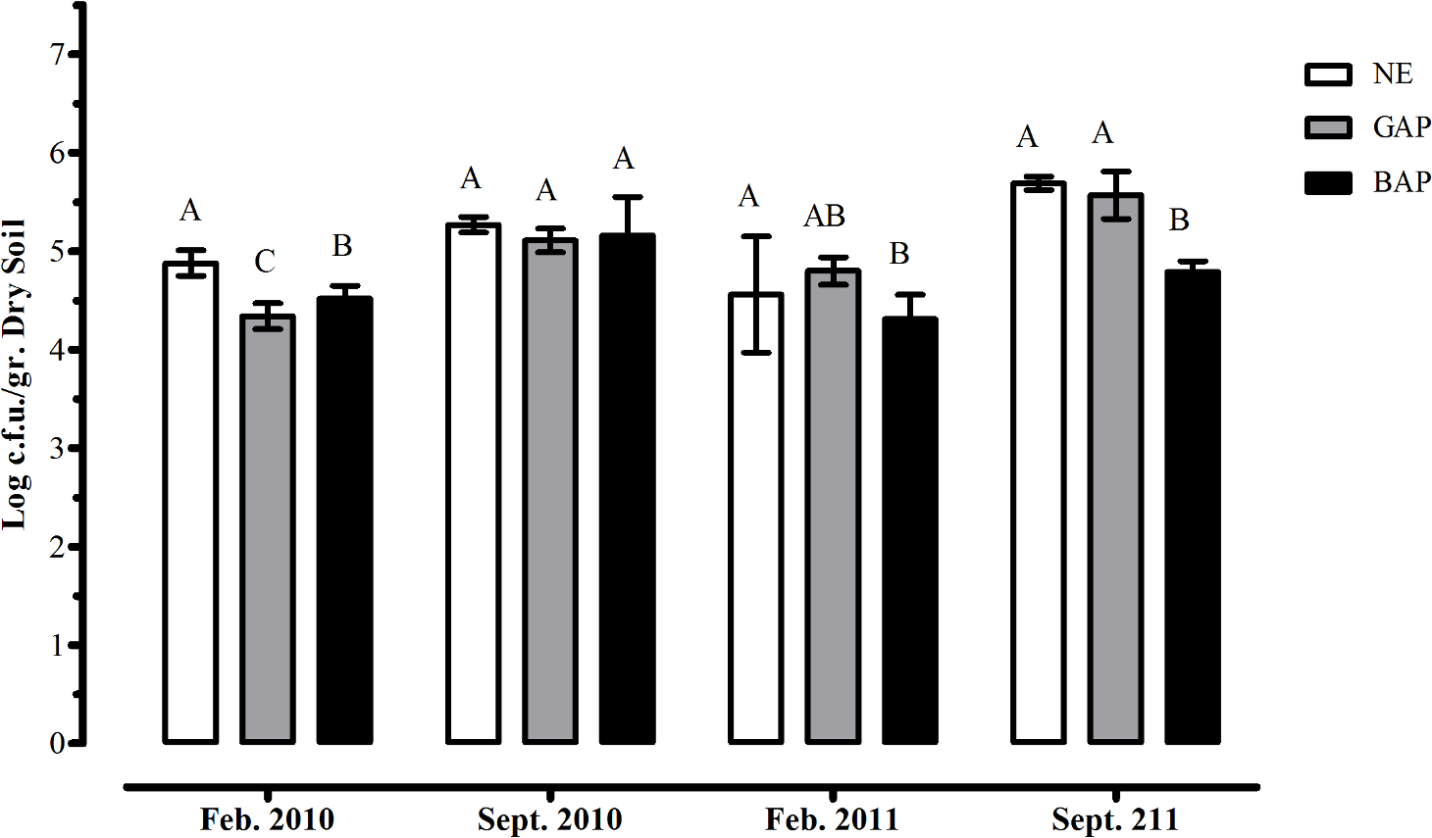
Quantification of *Burkholderia* species in Argentinian soils under contrasting agricultural management. Each group represents different time-scales where samples were obtained. Similar letters (A, B or C) do not differ at 5% level (LSD test, *p*<0.05).

The abundance of *Burkholderia* isolates was not significantly different among treatments across the sampling dates. Only non-farming Natural Environment treatments showed significant higher values in 3 out of 4 sampling dates (February 2010, 2011 and September 2011; Fig 1), thus reflecting the magnitude of the native *Burkholderia* population in the sampled soils under study. However, there was not a consistent pattern of bacterial counts in sampling sites subjected to contrasting agricultural management over the time. During the first year, only summer sampling (February 2010) showed significant differences between land uses, with BAP treatment showing higher *Burkholderia* counts, while winter sampling did not show statistical differences among treatments. During the second year a higher *Burkholderia* number in GAP treatments compared to BAP at both winter and summer seasons was observed. Therefore, the overall analysis showed that differences in similar treatments from different sampling seasons were never higher than 1 log c.f.u.g^-1^ dry soil, suggesting that *Burkholderia* abundance in sampled soils is evenly distributed, ranging 10^5^ c.f.u. per gram of dry soil regardless agricultural managements. It is worth mentioning that *Burkholderia* counts were not necessarily biased or influenced by the number of total culturable mesophilic heterotrophic bacteria, since parallel studies on the same samples showed that the amount of heterotrophic bacteria was similar for all sites and treatments, with an average of 6.2 log_10_ c.f.u g^-1^ [31,45] (S1 Fig).

#### Structure of *Burkholderia* population

**Fig 2:**
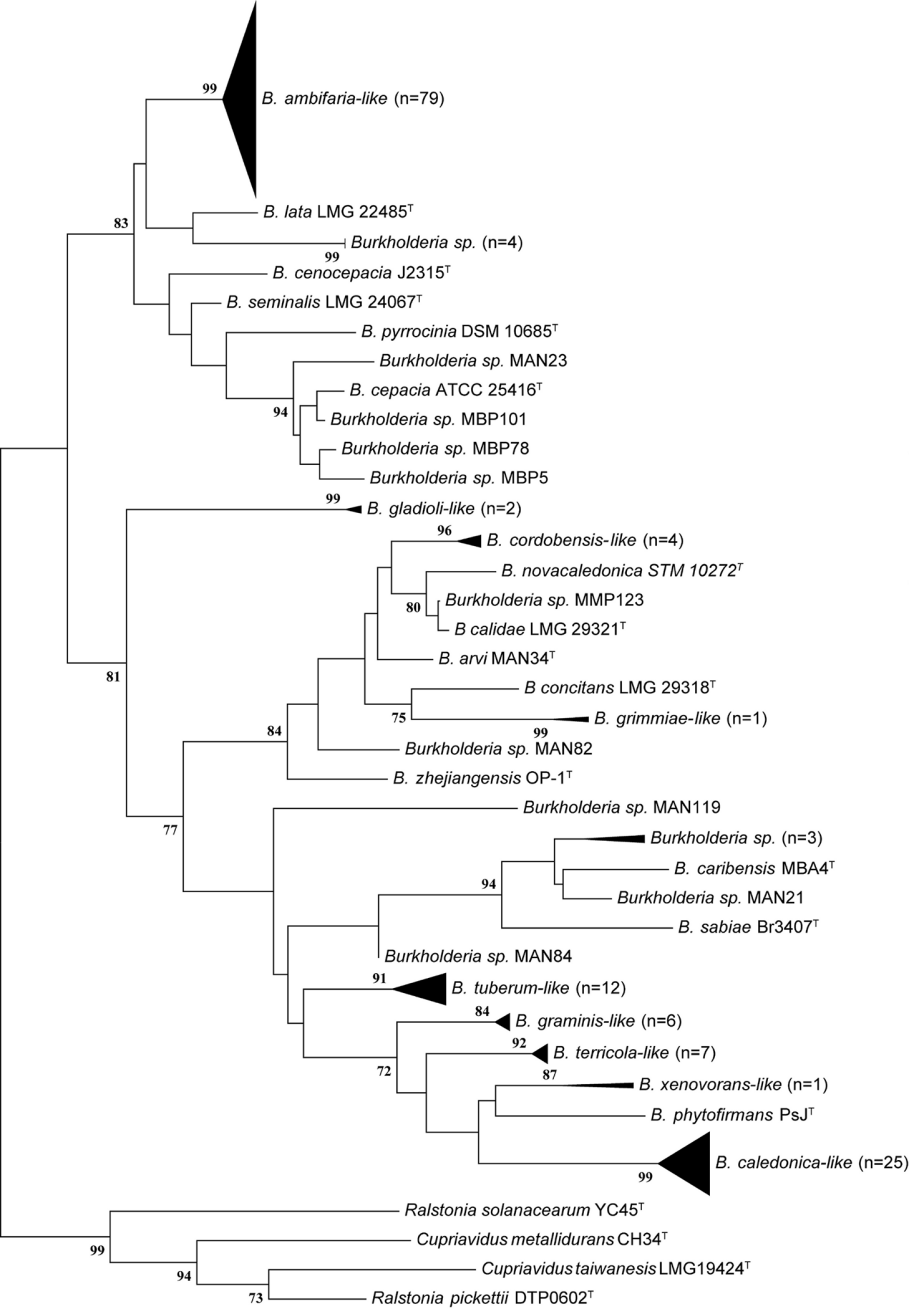
Phylogenetic analysis or *recA* sequences obtained from *Burkholderia spp*. The isolates were ascribed to known species of *Burkholderia* according to their clustering pattern with the type strains (indicated by the superscript ^T^) in highly supported clusters (bootstraps values ≥ 90%). This figure shows the identification of strains belonging to Bengolea site. Same analysis was done over the entire collection. *recA* sequences were deposited in the Genbank under the Accession numbers MF941496 to MF942066.

To determine the composition of the culturable *Burholderia* population in the different treatments at the species level, sequencing of the *recA* amplicon from all the 557 isolates was performed. In this sense, the phylogenetic information provided by the *recA* gene was sufficient for the identification of *Burkholderia* at the genus and species level. However, a more robust identification of strains belonging to the *Burkholderia* genus is carried out using higher resolution approaches such as the multilocus sequencing analysis of housekeeping genes, or the whole genome sequencing [37,46]. Therefore, the isolates were named as “taxon-*like*”, according to the position of each isolate in a booststrap-supported cluster (>90%) with the corresponding type strains (Fig 2). To test for the accuracy of this approach, we performed a multilocus sequencing typing (MLST) of random selected strains, and compared this phylogenetic assignment with the *recA* typing. MLST on the selected strains (four *B*. *ambifaria-like* strains, three *Burkholderia sp*.*-like* strains, and one *B*. *lata-like* strain) evidenced the same discriminatory power than the *recA* based approach (S2 Fig), validating the use to the *recA* gene for strain identification.

Regarding the previously discussed taxonomic criteria, we chose to keep the *Burkholderia* genus for all the isolates, assigning their taxonomic position according to the three described clades.

**Fig 3:**
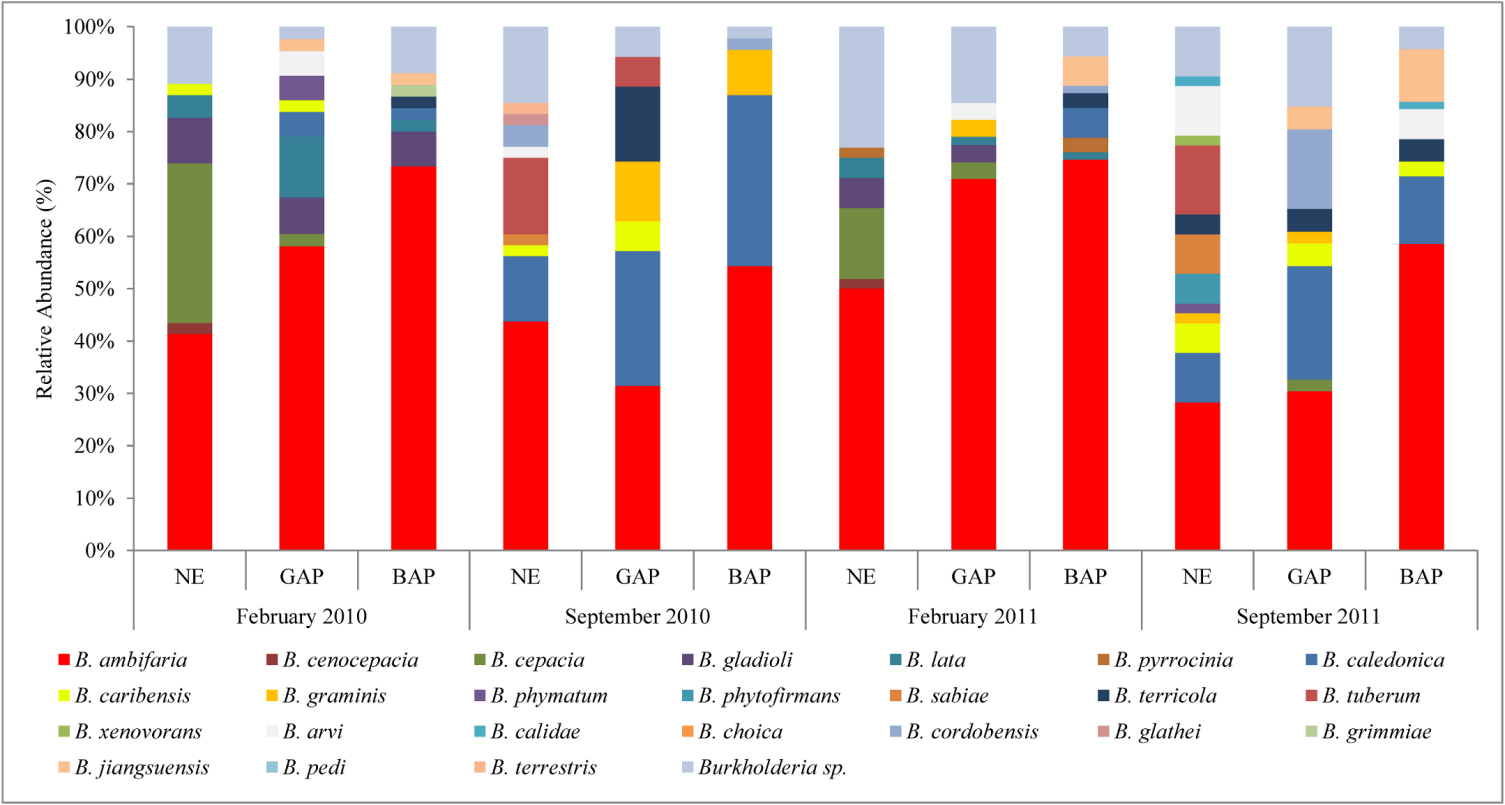
Structure of *Burkholderia* populations. Fig 3 shows the relative abundance of species composing each treatment at the four sampling dates. Each color represents different species according to the *recA* identification approach.

Thus, we identified 24 different *Burkholderia-like* species (Fig 3). The most abundant was *B*. *ambifaria*, which belongs to the BCC, representing the 51.3% of the whole strain collection. Within BCC we also found strains related to *B*. *cepacia*, *B*. *cenocepacia*, *B*. *lata*, *B*. *gladioli* and *B*. *pyrrocinia-like* species. Within PBE we observed the presence of isolates related to *B*. *caledonica*, *B*. *caribensis*, *B*. *phymatum*, *B*. *graminis*, *B*. *phytofirmans*, *B*. *sabiae*, *B*. *terricola*, *B*. *tuberum*, and *B*. *xenovorans-like* species, being *B*. *caledonica* and *B*. *caribiensis* the most abundant species, reaching up to 13% of total isolates. The less abundant species were those belonging to the BGG group: we observed the presence of strains related to *B*. *arvi*, *B*. *calidae*, *B*. *choica*, *B*. *cordobensis*, *B*. *glathei*, *B*. *grimmiae*, *B*. *jiangsuensis*, *B*. *pedi*, and *B*. *terrestris-like* species, representing *ca* 8% of the collection. Finally, 9.7% of the total strains could not be assigned to any recognized *Burkholderia* species, and were ascribed as *Burkholderia sp*. strains. As shown in Fig 3, the structure of the *Burkholderia* population varied across the different schemes imposed by the agricultural managements, a fact which led us to characterize the changing of the bacterial diversity according to the land use.

### Land use impact the *Burkholderia* population diversity

**Fig 4:**
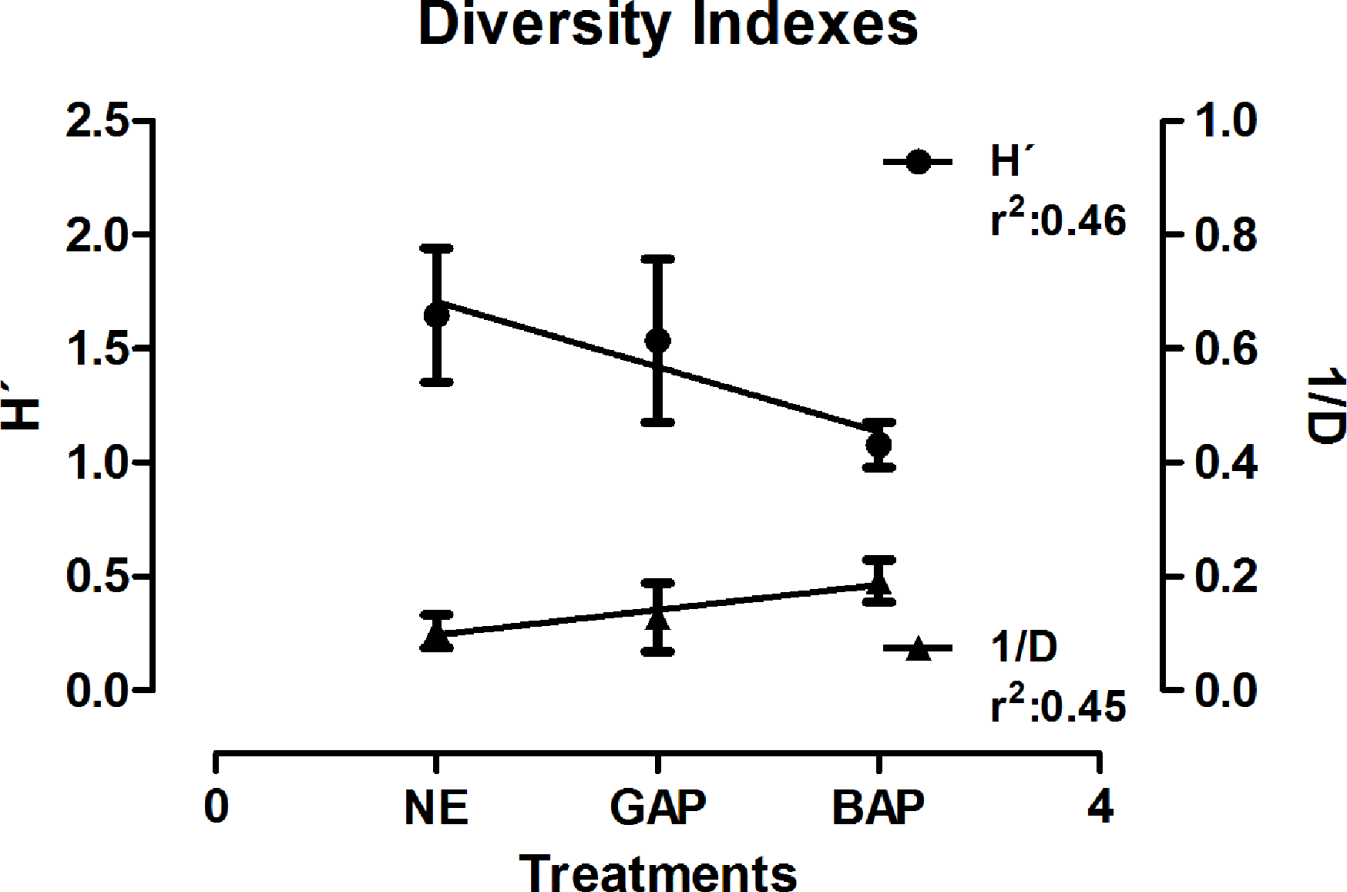
Evolution of biodiversity indexes according to land use. The evolution of Shannon (H´) and Simpson (1/D) indexes are shown, reflecting the influence of the soil managements on *Burkholderia spp*. diversity. Values obtained are the mean and standard deviations of treatments for each sampling date.

Alfa-biodiversity indexes were used to describe the variability of the *Burkholderia* population structure. Concerning this, we performed the analysis determining the evolution of the Shannon Index (H´) as an estimator of global diversity and the Simpson Index (1/D), reflecting the dominance of some species over the total population composition [47]. In order to unravel the main factor influencing the *Burkholderia* diversity and, concomitantly, the indexes determination, we performed a two-way Analysis of Similarity (ANOSIM) testing the significance of differences between groups of samples (regarding sites, treatments, and sampling dates). According to these results, sampling dates were the main factor influencing the biodiversity of *Burkholderia* samples. The analysis showed a statistically significant difference when sampling dates vs. treatments (R = 0.095; p< 0.04) and sampling dates vs. sites (R = 0.21; p < 0.0013) were analyzed, while the combination of the remaining factors (treatments vs sites, R = 0.07; p < 0.10) did not show a statistically valid comparison. In this regard, the regression analysis of the biodiversity index was accomplished on each sampling date for the three agricultural managements, which in parallel, allowed us to obtain an improved species coverage to determine unbiased diversity indexes (Table 2). Fig 4 shows the changing of diversity indexes across the different land uses. NE showed the largest biodiversity and the more evenness population structure according to the Shannon and Simpson indexes. In contrast, BAP showed the lowest values of biodiversity and the highest dominance values, while GAP treatments showed intermediates values in between NE and BAP treatments.

**Table 2:** Diversity values of *Burkholderia spp*. populations.

Treatments	Coverage (1-Singletons/s)	S(est) ± SD	Shannon Index (H´)	Simpson Index (1/D)
NE1	71.43	7 ± 1.54	1.48	0.29
GAP1	55.56	9 ± 1.54	1.46	0.37
BAP1	57.14	7 ± 1.2	1.02	0.55
NE2	55.56	9 ± 1.93	1.65	0.26
GAP2	87.50	8 ± 1.15	1.78	0.21
BAP2	80.00	5 ± 0.61	1.13	0.39
NE3	71.43	7 ± 1.14	1.4	0.33
GAP3	85.71	7 ± 0.7	1.06	0.51
BAP3	71.43	7 ± 1.14	0.97	0.57
NE4	72.73	11 ± 1.04	2.06	0.16
GAP4	77.78	9 ± 0.62	1.84	0.19
BAP4	83.33	6 ± 0.61	1.19	0.41

Diversity (H´, 1/D), richness (S) and coverage values for treatments at each sampling dates (1: February 2010; 2: September 2010; 3: February 2011; 4: September 2011).

Results showed an increase in dominance values in BAP respect to GAP and NE treatments. In order to evaluate changes in the *Burkholderia* species across treatments, we performed a SIMPER analysis. The analysis showed that *B*. *ambifaria-like* isolates increase their presence from NE to BAP treatments, with a twofold percentage increase for BAP treatments, and representing close to 40% of the *Burkholderia* strains isolated from this soil management (S2 Table). In addition, *B*. *caledonica-* and *B*. *jiangsuensis-like* isolates showed a higher presence in BAP treatments. In contrast, nine *Burkholderia*-*like* species were not present in BAP treatments, resulting in a loss of richness in soils subjected to this management. GAP treatments also showed nine non-detected species compared to NE treatments. However, the absent species in GAP comprised the least represented ones (*ca* 4% of contribution to total average population) with respect to BAP absent strains (*ca* 10% of contribution to total average population), which influenced differentially in the absolute values determined by the diversity indexes.

### Environmental factors affecting the diversity of *Burkholderia* populations

**Fig 5:**
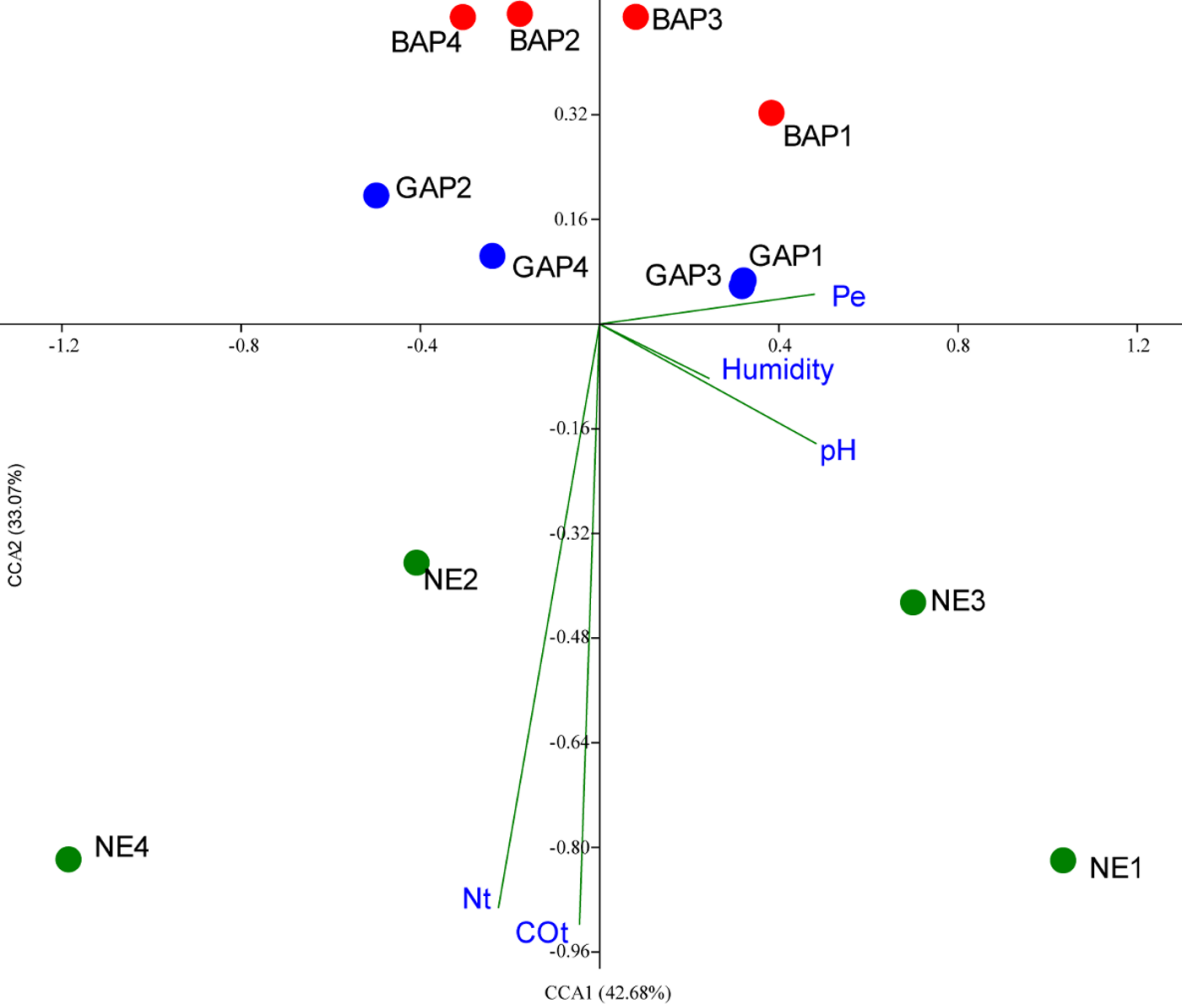
Multivariate analysis of *Burkholderia spp*. diversity according to soil properties. Lines indicate the magnitude of soil measured variables associated with bacterial community structures. Treatments are depicted as colored spots, and numbers indicate sampling dates (1: February 2010; 2: September 2010; 3: February 2011; 4: September 2011).

In order to determine the influence of soil chemical characteristics on the abundance of *Burkholderia*-like taxon, we performed a Canonical Correspondence Analysis (CCA) considering five edaphic factors: Total organic carbon (TOC), total nitrogen (Nt), extractable phosphorus (Pe), pH and soil humidity (S3 Table). This method allowed us to determine the variation in the abundance of species related to the measured soils variables. The global permutation test for all constraints together showed that the relation between the analyzed variables was significant (*p* = 0.016, based on 999 permutations). The first two components produced by the CCA accounted for 75.75% of the explained variance. As shown in Fig 5, the CCA plot shows a net separation of *Burkholderia* populations submitted to different land uses mainly by the total Nitrogen and total Organic Carbon content. This is indicated by the length of an environmental parameter line in the ordination plot, which displays the strength of the relationship of that parameter to community composition. The analysis showed that the soil composition, which is characterized by differential nutrient availability at each treatment, affected the structure of *Burkholderia* populations.

## Discussion

The use of no-till agricultural practices has been widely adopted by Argentinean farmers, reaching *ca* 80% of the total cultivated areas. The productivity advantages, as well as the economic benefits, are the main factors that made no-till agriculture so successful. Several studies reported on the effects that these agricultural practices have on soil biology, mainly focused in alterations of soil microbiota through metagenomics analysis [41,48,49,50,51]. The study of cultivable bacterial populations has been, however, unattended [31]. Even when this approach only works for a proportion of the whole soil microbiota, the culturability of a set of organisms allowed us not only to study the ecological aspects of this group but also to use of these organisms as a source of potential biotechnological products and biomarkers. In addition, biodiversity indexes showed enough sensitivity (to detect differences among treatments) as well as robustness (systematic behavior at different sampling points) which allows their use in the study of disruption of soil biodiversity by agricultural management system [52].

During 2009, a group of Argentinean scientists conformed the BIOSPAS, a consortium aiming at studying different aspects of the soil biology of agricultural sites subjected to differential management systems, all of them under a no-till scheme [53]. As members of the BIOSPAS Consortium, our first goal was to recognize the presence of cultivable *Burkholderia* species in crop productive soils as well as to unravel how its occurrence was influenced by different agricultural management methods. In this regard, we showed that culturable *Burkholderia* strains in agricultural soils reached slightly higher concentration than 1x10^5^ c.f.u.g^-1^ dry soil in natural environments. The *Burkholderia* concentration seems to be similar to previous results found in other soils. Using a similar approach, Pallud et al. showed the presence of 2.4x10^5^ c.f.u.g^-1^ of *Burkholderia* cells in soils from Southeast of France, while Salles et al. found lower concentrations of *Burkholderia* in acidic soils from The Netherlands, reaching 1x10^3^ c.f.u.g^-1^ of soil [26,33]. This last study, however, has not expressed the colony forming units counting in the same unities (dry weight soil), so the actual number could be slightly higher in those areas. The presence of the total *Burkholderia* population was affected by the agricultural management imposed on different sites. NE showed a higher presence of *Burkholderia* species but not in a regular pattern. It is broadly accepted that land use affect the presence and diversity of the soil microbiota, an effect that is especially remarkable when pristine sites are derived for agricultural purposes [54,55,56]. However, *Burkholderia* species and particularly BCC species -the most abundant in our case-were characterized as r-strategists organisms [12,57]. Even when suffering high mortality rates, r-strategists organisms achieve higher growth rates, which confer them with the ability to rapidly occupy disrupted ecological niches [58]. These characteristics could contribute with a high turnover in *Burkholderia* population species, keeping in this way different steady states in the soil cells density according to the stress factors imposed on the habitat. This high turnover in the number of *Burkholderia spp*. could mask the effects of soil management, making their quantification not a valid measure for studying the impact of soil treatments on microbiological diversity.

Biodiversity Indexes clearly showed the effect that agricultural managements had on the *Burkholderia* structure population. The decrease of diversity of *Burkholderia* species, through the negative correlation in the Shannon Indexes, as well as the increase in the dominance values reflect the effect of BAP treatments on *Burkholderia* population structure. Therefore, we found strong evidence showing that the agricultural management impacts *Burkholderia* diversity on soils. Salles and colleagues showed changes in *Burkholderia* composition in experiments carried out in soils with different land history. They showed the effects that different agricultural practices, such as tillage, crop rotation, or fertilization had on *Burkholderia* biodiversity, showing that grassland sites usually preserve the most diverse environment in soils [26,27,59]. Our experimental design comprised sites with agricultural crop production under no-tillage schemes, which remove a key environmental variable responsible of diversity changes previously observed in *Burkholderiales* [60]. Even with a more sustainable soil use, the agricultural scheme imposed on BAP treatments—with mono-cropping, high doses of pesticides and unbalanced fertilization—was deleterious to maintain the diversity of *Burkholderia* species in our agricultural soils, showing the increase in the dominance of *B*. *ambifaria-* like strains and the absence of the less abundant *Burkholderia* species when soils were subjected to non-sustainable land use. Similar results were observed in the same experiments for cultivable *Pseudomonas spp*. populations, with low diversity structures on GAP and BAP treatments, with BAP showing the lowest diversity [31]. Regarding treatments, results showed that no-till schemes *per se* are not sufficient to maintain a richer soil microbiota, and additional traits should be considered (e.g.: doses of pesticides, balanced fertilization, crop rotation) when sustainability of productive soils is a goal to fulfil in productive agricultural schemes.

Analysis of the influence of soil composition on *Burkholderia* population diversity showed that biodiversity was mainly linked to Total Organic Carbon and Total Nitrogen in soils. Similar results were observed when the global bacterial diversity was analyzed on the same samples in a metagenomic study [41]. In different soils, it has been shown that the presence of *Burkholderia* species is correlated with their ability to degrade cellulose as a carbon source [61,62], and their presence is relevant to maintain soil organic carbon concentration through their cellulolytic activity [63]. In fact, previous analyses on the same soils samples of our study have shown that GAP treatments improve the soil organic carbon compared to BAP treatments, especially for the labile organic fractions [44,64]. Previous findings have shown that soil pH is a main determining of the *Burkholderia* spp. abundance across soils [65]. In our case, pH was also an important soil factor but not the main one. This could be a result of a lack of contrasting pH values across sites (media pH value = 6.13±0.3), a soil pH range in which the higher abundance of *Burkholderia* spp. was found [65]. Therefore, it is plausible to propose that, in our conditions, land uses that improve the soil organic content could maintain a more diverse structure of *Burkholderia* populations through a better availability of carbon sources.

With a rising population worldwide, food production under sustainable schemes is necessary to preserve natural resources. As part of the soil microbiota, the diversity of *Burkholderia* species is affected by land use even in a no-till crop system, addressing the necessity to perform sustainable agricultural schemes for crop production in order to avoid the loss of bacterial species. This is important not only from an ecological point of view, but also for crop productivity, since a low bacterial diversity could interfere in natural processes that actually occur in soils, as nitrogen fixation or natural competition against pathogenic microorganism, which support crop productivity and grains yields in a sustainable manner [66].

## Supporting information

S1 Fig Map of the geolocated sampling sites template map downloaded from http://www.google.com/earth/. (TIFF)

S2 Fig Quantification of *Burkholderia* spp. in relation with *total Heterotrophes* in Argentinian soils under contrasting agricultural management. Each group represents different time-scales where samples were obtained. Similar letters (A, B or C) do not differ at 5% level (LSD test, p<0.05).
(TIF)

S3 Fig Multilocus sequencing typing of selected *Burkholderia spp*. strains. The phylogenetic tree shows the phylogenetic position of selected strains supporting the *recA* based approach. *B*. *ambifaria* strains are marked with black circles, while remaining strains (*B*. *lata* and *Burkholderia spp*.) are marked with a black triangle. The phylogenetic tree was built with the concatenated sequences of *Burkholderia* Type Strains of the 7 housekeeping genes, obtained from the publicly available database. The evolutionary history was inferred using the Neighbor-Joining method [1]. The optimal tree with the sum of branch length = 2.31961162 is shown. The percentage of replicate trees in which the associated taxa clustered together in the bootstrap test (1000 replicates) is shown next to the branches [2]. The tree is drawn to scale, with branch lengths in the same units as those of the evolutionary distances used to infer the phylogenetic tree. The evolutionary distances were computed using the Tamura 3-parameter method [3] and are in the units of the number of base substitutions per site. The rate variation among sites was modeled with a gamma distribution (shape parameter = 5). The analysis involved 66 nucleotide sequences. All positions with less than 95% site coverage were eliminated. That is, fewer than 5% alignment gaps, missing data, and ambiguous bases were allowed at any position. There were a total of 2760 positions in the final dataset. Evolutionary analyses were conducted in MEGA6 [4]. Gene sequences were deposited in the Genbank under the Accession number MF942067—MF942074 for *atpD*, MF942075—MF942083 for *gltB*, MF942084—MF942092 for *gyrB*, MF942093—MF942101 for *lepA*, MF942102—MF942110 for *phaC*, MF942111—MF942119 for *recA*, and MF942120—MF942127 for *trpB*.
(TIF)

S1 Table Values of total culturable *Burkholderia spp*. present at each treatment according to sampling dates. Values are mean of and variance of the log_10_ of colony forming units, expressed in dry soils weight (c.f.u.gr^-1^ soil dry weight; 1: February 2010; 2: September 2010; 3: February 2011; 4: September 2011).
(PDF)

S2 Table SIMPER analysis of variation in the *Burkholderia* population structures according to the soil management system. Mean abundance values are expressed as percentages (%).
(PDF)

S3 Table Soil chemical variables used in the canonical corresponding analysis. COt: total organic carbon, g kg-1; Nt: total Nitrogen (g kg-1); Pe: extractable phosphorous (mg. kg-1); Humidity: percentage (%). Values were obtained from Duval y col. [64]. Number indicate sampling dates (1: February 2010; 2: September 2010; 3: February 2011; 4: September 2011).
(PDF)
