# Supplementary figures and images for: Biodiversity of cultivable Burkholderia species in Argentinean soils under no-till agricultural practices
Source: PLoS One. 2018 Jul 12;13(7):e0200651. doi: 10.1371/journal.pone.0200651 (PMC6042781; doi:10.1371/journal.pone.0200651)

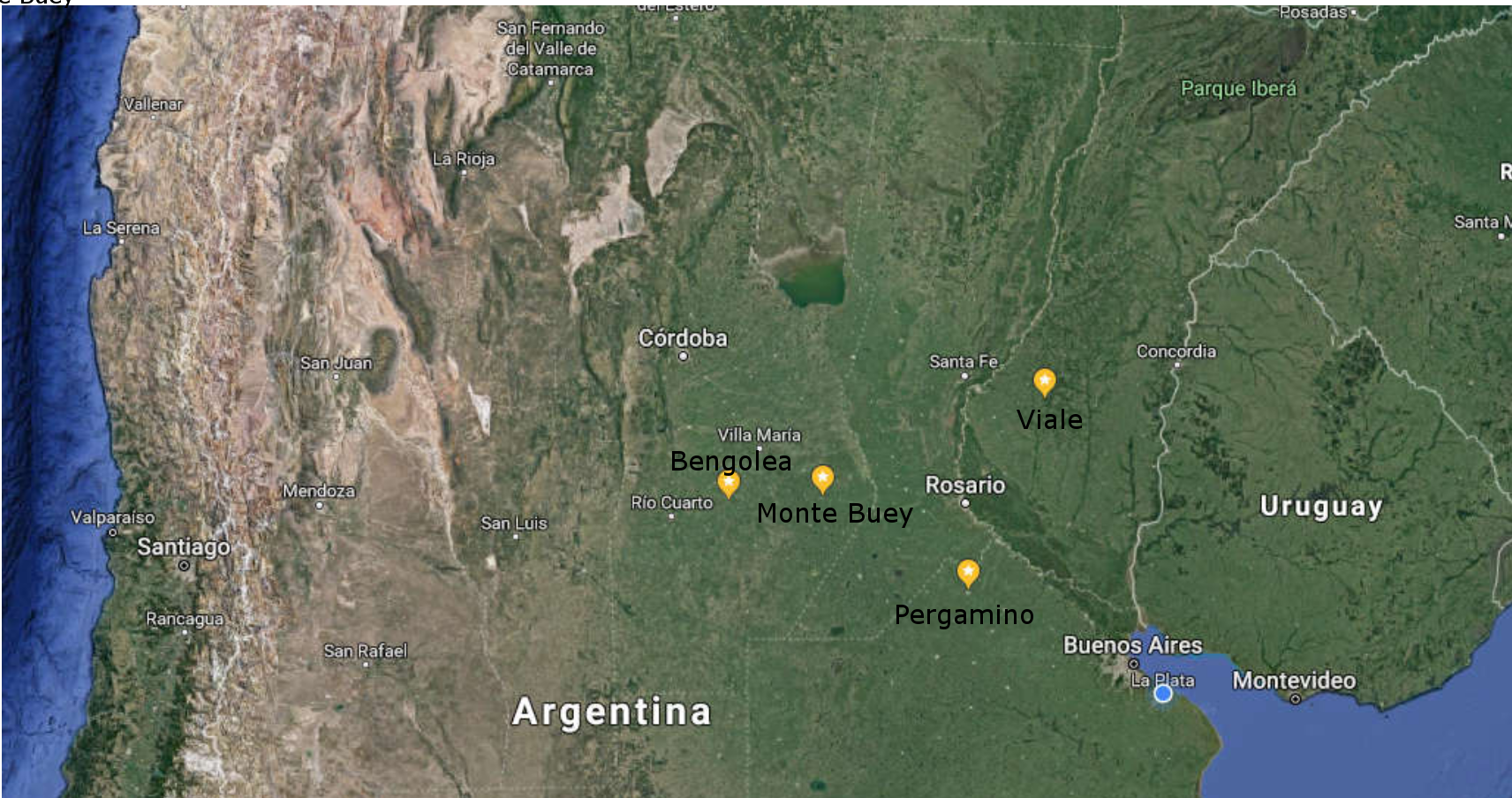

Supplement: S1 Fig — (TIFF) [file pone.0200651.s001.tiff]

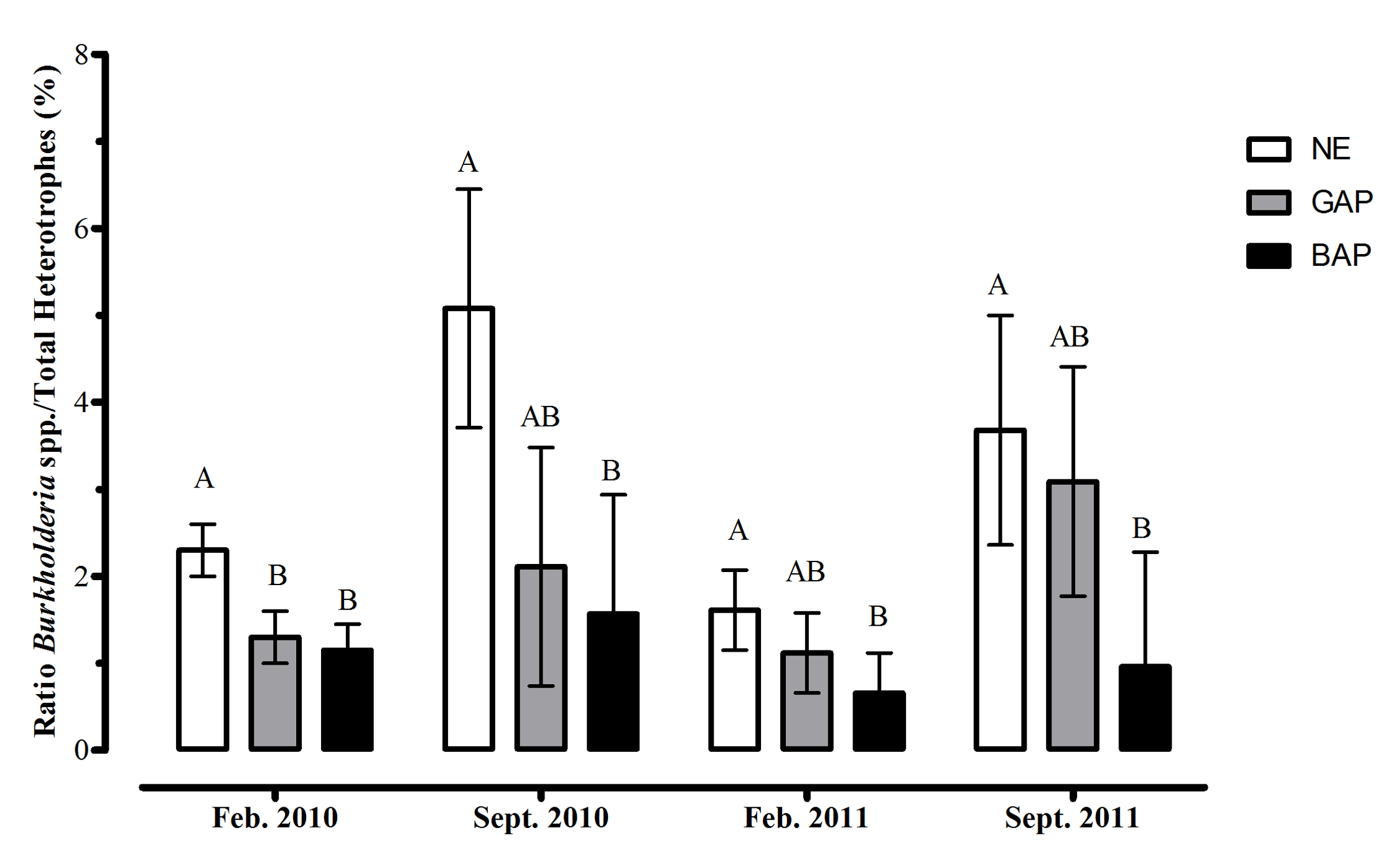

Supplement: S2 Fig — Each group represents different time-scales where samples were obtained. Similar letters (A, B or C) do not differ at 5% level (LSD test, p<0.05). (TIF) [file pone.0200651.s002.tif]

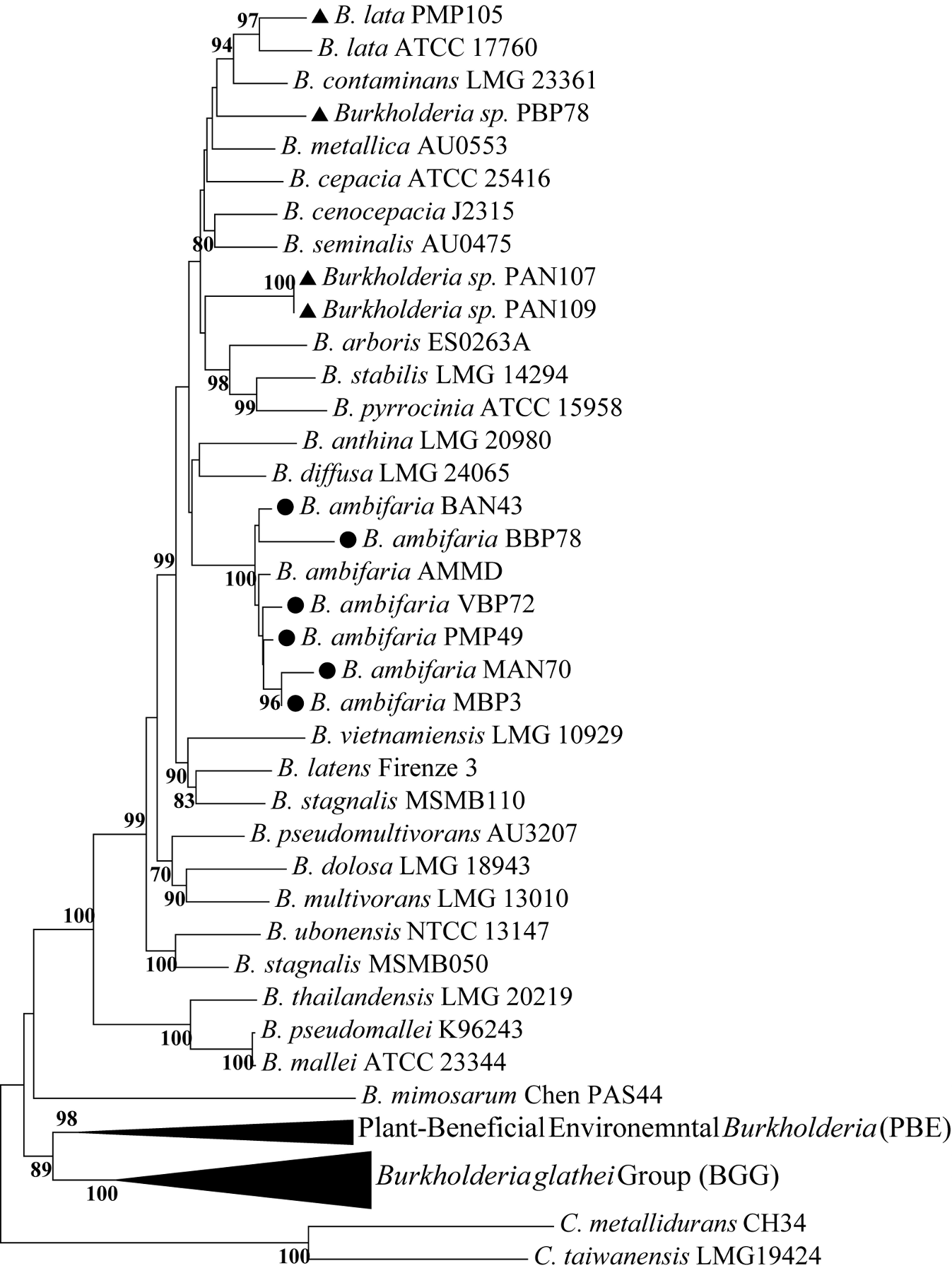

Supplement: S3 Fig — The phylogenetic tree shows the phylogenetic position of selected strains supporting the recA based approach. B. ambifaria strains are marked with black circles, while remaining strains (B. lata and Burkholderia spp.) are marked with a black triangle. The phylogenetic tree was built with the concatenated sequences of Burkholderia Type Strains of the 7 housekeeping genes, obtained from the publicly available database. The evolutionary history was inferred using the Neighbor-Joining method [1]. The optimal tree with the sum of branch length = 2.31961162 is shown. The percentage of replicate trees in which the associated taxa clustered together in the bootstrap test (1000 replicates) is shown next to the branches [2]. The tree is drawn to scale, with branch lengths in the same units as those of the evolutionary distances used to infer the phylogenetic tree. The evolutionary distances were computed using the Tamura 3-parameter method [3] and are in the units of the number of base substitutions per site. The rate variation among sites was modeled with a gamma distribution (shape parameter = 5). The analysis involved 66 nucleotide sequences. All positions with less than 95% site coverage were eliminated. That is, fewer than 5% alignment gaps, missing data, and ambiguous bases were allowed at any position. There were a total of 2760 positions in the final dataset. Evolutionary analyses were conducted in MEGA6 [4]. Gene sequences were deposited in the Genbank under the Accession number MF942067—MF942074 for atpD, MF942075—MF942083 for gltB, MF942084—MF942092 for gyrB, MF942093—MF942101 for lepA, MF942102—MF942110 for phaC, MF942111—MF942119 for recA, and MF942120—MF942127 for trpB. (TIF) [file pone.0200651.s003.tif]
