# Supplementary material for: Biodiversity of cultivable Burkholderia species in Argentinean soils under no-till agricultural practices
Source: PLoS One. 2018 Jul 12;13(7):e0200651. doi: 10.1371/journal.pone.0200651 (PMC6042781; doi:10.1371/journal.pone.0200651)
Supplement: S1 Table — Values are mean of and variance of the log10 of colony forming units, expressed in dry soils weight (c.f.u.gr-1 soil dry weight; 1: February 2010; 2: September 2010; 3: February 2011; 4: September 2011). (PDF) [file pone.0200651.s004.pdf]

Treatments	Variable	Mean	Variance
NE1	February 2010	5.88	0.11
NE2	September 2010	5.27	0.08
NE3	February 2011	4.56	0.59
NE4	September 2011	5.69	0.07
GAP1	February 2010	5.34	0.08
GAP2	September 2010	5.12	0.12
GAP3	February 2011	4.80	0.14
GAP4	September 2011	5.58	0.24
BAP1	February 2010	5.52	0.03
BAP2	September 2010	5.16	0.39
BAP3	February 2011	4.31	0.25
BAP4	September 2011	4.79	0.11
