# Supplementary material for: Biodiversity of cultivable Burkholderia species in Argentinean soils under no-till agricultural practices
Source: PLoS One. 2018 Jul 12;13(7):e0200651. doi: 10.1371/journal.pone.0200651 (PMC6042781; doi:10.1371/journal.pone.0200651)
Supplement: S2 Table — Mean abundance values are expressed as percentages (%). (PDF) [file pone.0200651.s005.pdf]

Taxon	Av. dissim	Contrib. %	Cumulative %	Mean abund. NE	Mean abund. GAP	Mean abund. BAP
B. ambifaria	14.97	31.93	31.93	20.3	23.5	38
B. caledonica	5.55	11.84	43.78	2.75	5.25	7.25
Burkholderia sp.	3.904	8.331	52.11	7.25	4.75	3
B. cepacia	3.851	8.219	60.33	5.25	1	0
B. tuberum	2.49	5.314	65.64	3.5	0.5	0
B. jiangsuensis	1.934	4.128	69.77	0	0.75	3
B. terricola	1.697	3.622	73.39	0.5	1.75	1.5
B. arvi	1.625	3.467	76.86	1.5	1	1
B. gladioli	1.619	3.454	80.31	1.75	1.25	0.75
B. graminis	1.594	3.402	83.72	0.25	1.75	1
B. cordobensis	1.586	3.385	87.1	0.5	1.75	0.5
B. lata	1.434	3.061	90.16	1	1.5	0.5
B. caribensis	1.163	2.482	92.64	1.25	1.25	0.5
B. sabiae	0.8111	1.731	94.37	1.25	0	0
B. phytofirmans	0.4817	1.028	95.4	0.75	0	0
B. phymatum	0.4646	0.9915	96.39	0.25	0.5	0
B. pyrrocinia	0.4092	0.8732	97.27	0.25	0	0.5
B. cenocepacia	0.3345	0.7139	97.98	0.5	0	0
B. calidae	0.2684	0.5726	98.55	0.25	0	0.25
B. grimmiae	0.1801	0.3843	98.94	0	0	0.25
B. terrestris	0.1688	0.3603	99.3	0.25	0	0
B. glathei	0.1688	0.3603	99.66	0.25	0	0
B. xenovorans	0.1606	0.3427	100	0.25	0	0
