# Supplementary material for: Biodiversity of cultivable Burkholderia species in Argentinean soils under no-till agricultural practices
Source: PLoS One. 2018 Jul 12;13(7):e0200651. doi: 10.1371/journal.pone.0200651 (PMC6042781; doi:10.1371/journal.pone.0200651)
Supplement: S3 Table — COt: total organic carbon, g kg-1; Nt: total Nitrogen (g kg-1); Pe: extractable phosphorous (mg. kg-1); Humidity: percentage (%). Values were obtained from Duval y col. [64]. Number indicate sampling dates (1: February 2010; 2: September 2010; 3: February 2011; 4: September 2011). (PDF) [file pone.0200651.s006.pdf]

	COt	pH	Nt	Pe	Humidity
AN1	2.87	6.68	0.22	28.01	24.87
AN2	2.89	5.92	0.23	7.96	15.83
AN3	3.02	5.91	0.24	11.98	17.6
AN4	3.03	6.05	0.27	14.1	17.26
GAP1	2.11	6.59	0.17	37.61	26.09
GAP2	2.37	5.96	0.19	15.2	23.85
GAP3	2.29	5.81	0.19	19.57	14.49
GAP4	2.34	6.04	0.2	21.74	19.39
BAP1	1.74	6.6	0.13	27.15	15.96
BAP2	1.9	5.89	0.15	13.6	20.24
BAP3	1.94	6.04	0.15	13.85	22.41
BAP4	1.8	6.03	0.16	14.49	13.69
